# Evaluating the Intrinsic Electromagnetic Field Generated by Neurons From Repetitive Motor Activities in Humans With a Non-contact Non-invasive Electromagnetic Helmet

**DOI:** 10.7759/cureus.23006

**Published:** 2022-03-09

**Authors:** James Brazdzionis, James Wiginton, Tye Patchana, Paras Savla, James Hung, Yongming Zhang, Dan E Miulli

**Affiliations:** 1 Neurosurgery, Riverside University Health System Medical Center, Moreno Valley, USA; 2 Electrical Engineering, Quasar Federal Systems, San Diego, USA; 3 Medical Physics, Quasar Federal Systems, San Diego, USA; 4 Neurosurgery, Arrowhead Regional Medical Center, Colton, USA

**Keywords:** neuroimaging, new technologies in neurosurgery, neurosurgery, motor, magnetic field sensing, magnetic field, electromagnetic field

## Abstract

Introduction

The actions of neurons are dependent on electrochemical signal pathways mediated by neurotransmitters and create measurable electrical charges. These charges have been found to be measurable through neuroimaging technologies and now through a novel non-contact non-invasive sensor without supercooling. Identifying whether this technology can be appropriately interpreted with synchronized motor well-defined activities in vivo may allow for further clinical applications.

Methods

A non-contact, non-invasive helmet constructed and modified using shielding technology with proprietary magnetic field sensors was utilized to measure the brain’s electromagnetic field (EMF). Human volunteers donned helmets and were asked to perform repetitive tapping exercises in order to identify waves consistent with tapping from the left and right hemispheres. A gyroscope was utilized to ensure that measured waves were not from micro-movement but were from neuronal firing. Multiple individuals were tested to evaluate the reproducibility of tapping and commonalities between individuals

Results

Right and left-sided tapping generated discernible wave changes from baseline measurements obtained by the helmet without a subject as well as differed from when the subject was at rest. Wave patterns varied from person to person but were overall similar in each subject individually. Shielding was necessary to identify signals but EMF was identified when shielding was transitioned from around the helmet to within the helmet design.

Conclusion

It is possible to measure in-vivo electromagnetic fields generated by the human brain generated by stereotyped tasks in a non-contact non-invasive manner. These waves were reliably obtained within each individual with some variability in morphology from subject to subject however were similar in each subject. Signals varied based on activity and stereotyped motor activities were identified. A helmet using shielding technology within the helmet itself was able to effectively identify EMF signals. Future analysis may focus on translating these waves into functional mapping for clinical applications.

## Introduction

Human thought processes and activities are generated from the brain using a complex balance of electrochemical firing and signal transduction generated by neurons. Neurons themselves maintain a standard resting membrane potential of -60 mV [1]. When these neurons reach a threshold potential, voltage-gated sodium channels open driving positively charged sodium ions into the cell, and when a threshold is reached these sodium channels close and voltage-gated potassium channels open driving potassium out of the cell down an electrochemical gradient driving the cell into a hyperpolarized state below resting membrane potential [2]. This stereotyped ion-mediated firing creates significant polarity and electrical changes within each neuron during each action potential. These action potentials work in a summative fashion to allow for larger signal transduction and higher cortical activities. Due to the creation of an electrical charge a magnetic field is generated.

It has been found by Wiginton et al. that this magnetic field can be measured in a non-invasive fashion using sensitive sensor technologies [3]. Accordingly, if this magnetic field can be measured it may also be correlated to normal activities of brain function. One such well-documented activity is the motor function which is well known to have dominant function derived from the motor cortex of the contralateral frontal lobe [2]. These motor activities have been documented in magnetoenceophalography (MEG) and electroencephalography (EEG); however, we wished to understand the possibility of measuring these activities using room temperature, non-invasive electromagnetic sensors mounted to helmet among volunteer participants to identify if these stereotyped motor activities were reliably identifiable, varied in sidedness, and varied from person to person [4].

## Materials and methods

The investigation proceeded with the Institutional Review Board (IRB) approval (approval number 21-05) at Arrowhead Regional Medical Center, Colton, CA. Initial testing was conducted using a non-shielded helmet and transitioned to use with a shielded helmet.

Non-shielded helmet

Using a helmet with mu-metal shielding, copper mesh, and four Bx, By, Bz, and B319 proprietary electromagnetic passive sensors (QUASAR Federal Systems, San Diego, CA), electromagnetic fields (EMFs) of volunteer participants were identified and measured as described previously by Wiginton et al. [3]. These sensors were then configured within a shielded helmet with sensors oriented towards the motor strip based on anatomical landmarks. The proprietary sensors demonstrated a detection sensitivity of 1 pT/rtHz at 1 Hz with responses captured between 1 Hz and 2 kHz. The utilized sensors were 18 inches long by 3/4 inches in diameter. Sensors are connected to amplifiers and were configured with a 10X gain/2 kilohertz gain/filter module as configured by Wiginton et al. [3].

Results were captured at 5000 Hz on a laptop using a National Instruments Data Acquisition Card and LabVIEW software and post-processed using Igor® Pro 8 software (Wavemetrics, Inc Lake Oswego, OR). The time-domain data was transformed into the frequency domain using FFT (Fast Fourier Transfer), the cumulative composite of the voltage recorded in 20-second bins versus frequency recorded over 120 seconds. Data was binned into 0.3 Hz from 1 Hz to 2 kHz for every 20 seconds.

A reproducible synchronizing stimulus protocol was developed for all tests and subjects. The synchronization occurred to sound from a background metronome (non-ferromagnetic) outside of the Faraday cage, producing clicks at 120 bpm (2 Hz) to allow for detecting reproducible brain activity recordings from the subject while synchronizing tapping of hands and/or feet. The sensors were consistently positioned with the positive end (+) towards the scalp. During tests, the sensor angle to the brain surface was changed after a test was completed. Sensor distance from the scalp was changed from touching to 1.5 cm, 3.0 cm, and 4.5 cm away. Tapping of the hand was done by lifting the hand as one unit where the wrist remained on the ground and tips of the fingers were elevated 6.6 cm. Tapping of the foot was done with the knees and hips bent by lifting the foot as one unit where the heel remained on the ground and the tips of the toes were elevated 6.6 cm. Graphs were plotted using this binned data and FTT to rest and activity data where rest is designated as the activity sensed by the sensor when the designated named activity is not occurring. This rest-activity should correspond to baseline brain activity without the added motor stimulus.

**Figure 1:**
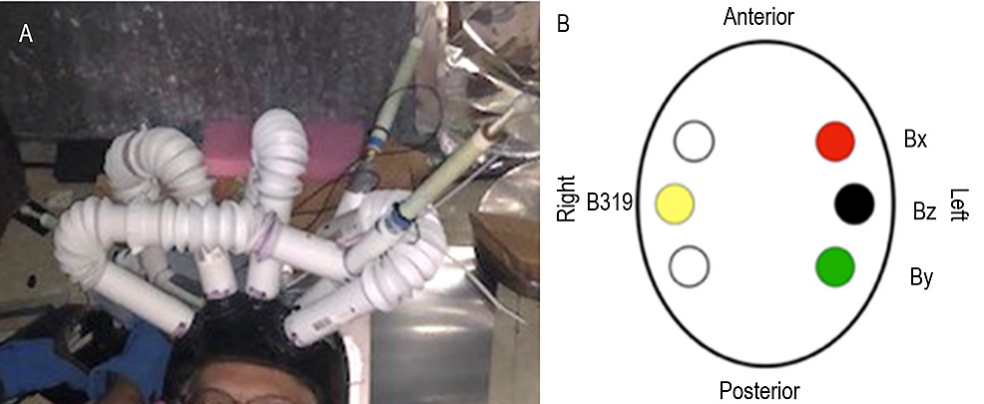
Sensor positioning using the initial non-shielded helmet Panel A identifies sensor position on a human subject. Sensors were oriented with a sensor in the left frontal region (sensor Bx), near the left motor strip (Bz), and posteriorly in the left parietal region (sensor Bz). On the right side, a sensor (sensor B319) was located across from the left Bz sensor approximating the right motor strip. Panel B identifies a graphical representation of sensor positioning (with color-coding based on graph results). B319: sensor B31; Bx: sensor Bx; Bz: sensor Bz; By: Sensor By

An initial helmet was constructed to allow the exact positioning of sensors in relation to the brain topography and therefore function (Figure 1). The plastic unshielded sensor tubes allowed the sensors to be adjusted so that they were either in direct contact with or at a measured distance from (1.5 cm, 3.0 cm, and 4.5 cm) the scalp. The sensor’s ability to record large amounts of data makes interpretation difficult without a place to look. To identify the sensor recorded response, a time-linked 2 Hz specific audio stimulus from a metronome was used to synchronize movement and allow better discrimination from background brain activity. A gyroscope was also attached to the helmet to record any movement and when it occurred during the recording. Angles of the sensors were defined as below in Table 1. During testing with this initial helmet, a mu-metal shield was draped over the helmet within a faraday cage.

**Table 1:** Angles of sensor positioning Angles of sensor positioning using the non-shielded helmet are represented for each plane

Plane	Sensor Bx	Sensor B319	Sensor By	Sensor Bz
Axial	-24°	-11°	-14°	-12°
Sagittal	-18°	-10°	18°	13°
Coronal	-11°	3°	10°	0°

Tests were performed on the volunteer human subject employing a set of templated activities. These included an initial 30 second (s) break, 60 s of right-hand tapping, 30 s of break, 60 s of left-hand tapping, 30 s of break, 60 s of right-foot tapping, 30 s of break, 60 s of left-foot tapping, 30 s of break, 60 s of right-sided hand and foot-tapping, 30 s of break, 60 s of left-sided hand and foot-tapping, 30 s of break, 60 s of tapping of bilateral hands and feet, and finally 30s of break. Tapping occurred using the reproducible protocol above.

Sensor repositioning

**Figure 2:**
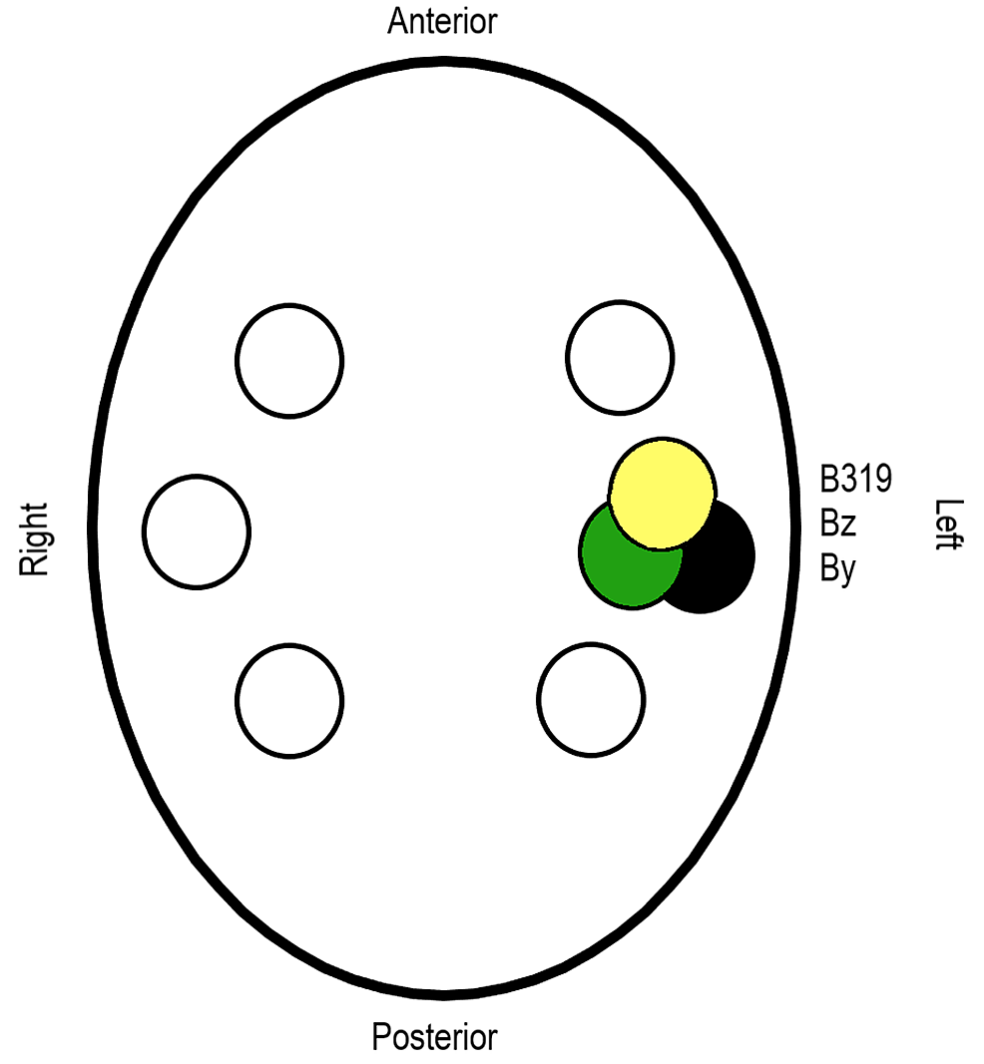
Repositioned sensors Graphical representation of sensor repositioning for additional tests in the non-shielded helmet. B319: Sensor B319; Bz: Sensor Bz; By: Sensor By

Sensors were repositioned as below in Figure 2. Testing was completed using a subject completing right-hand tapping, left-hand tapping, right and left foot-tapping while tapping with both the upper and lower hands and feet on the right and left sides and while tapping with all extremities. Rest was also recorded. All activities were completed using the standardized tapping protocol. Sensors were repositioned within the sagittal, coronal, and axial planes during these activities to assess for differences in the ability to sense the EMF based on orientation. Bx and Bz sensors were recorded and positioned as below in Table 2.

**Table 2:** Angles of repositioned sensors Placement angles are represented for repositioned sensors in axial, sagittal and coronal planes

Plane	Sensor Bx	Sensor Bz	Sensor B319
Axial	-5°	2°	-11°
Sagittal	2°	-7°	-10°
Coronal	14°	3°	3°

Shielded helmet

**Figure 3:**
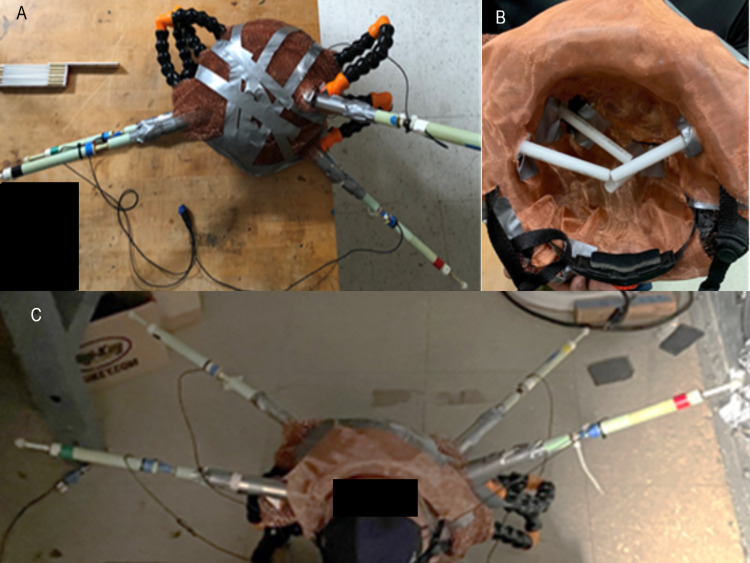
Prototype shielded helmet A prototype shielded helmet used in testing is represented. Panel A represents an external view. Panel B represents the interior of the helmet with piping placed to represent the electromagnetic field column sensed by each sensor. Panel C identifies the helmet on a subject's head.

The helmet was modified by adding shielding to improve sensitivity and reduce external noise as pictured below in Figure 3. The helmet was constructed using two layers of mu-metal 2.5 cm apart with inner and outer layers of interlaced copper mesh to attempt to absorb and reflect EMF. Channels were placed within the helmet for sensors to pass through.

Further tests were completed using the updated shielded helmet with sensors focused on the left motor strip. Hand or foot tapping was initially at a rate of 2 Hz, twice per second. Then completed at 3Hz and 3.467 Hz using the same protocol as above to determine if the recorded EMF signal was unique for the task and from the individual. The different signals also permitted the identification of different areas of the brain producing the EMF.

Shielded helmet with multiple subjects

Seven volunteers participated in repeating tapping activities to evaluate reproducibility and reliability of the ability to evaluate tapping amongst subjects. These participants were healthy subjects, over the age of 18, with no known intracranial pathology. Sex distribution consisted of two female volunteers and five male volunteers. Subjects were asked to lie supine in a Faraday cage without light or stimulation within the room. After donning the helmet with sensors directed at the left motor strip, the right hand was tapped of the hand using the tapping protocol. This was repeated with each subject to obtain reliable data. Prior to measuring, volunteers attempted this stereotyped activity to ensure they completed the activity with appropriate timing and without lifting the hand off the ground.

## Results

Evaluation of optimal positioning of sensors

**Figure 4:**
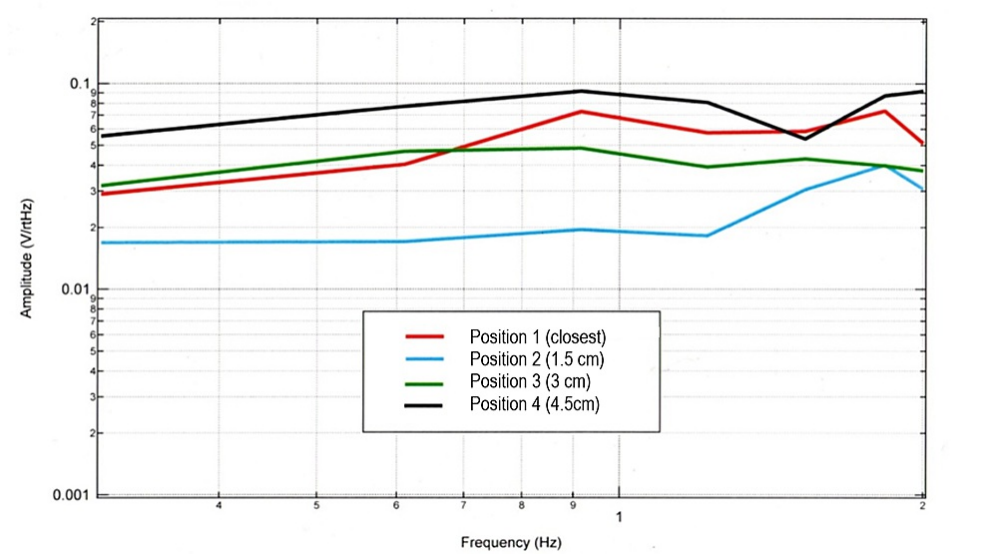
Amplitude of sensor B319 demonstrating effects of distance away from the measured electromagnetic field during tapping exercise The Fourier transformed data of frequency versus amplitude were plotted for sensor B319 when tapping at 2 Hz at different distances between the subject and sensor. cm: Centimeters; V/rtHz: Voltage divided by square route of Hertz; Hz: Hertz

As preliminary testing identified that it was possible to sense cortical EMF activity using these proprietary sensors [3]. Initial testing was attempted to investigate if there was a baseline optimal position for testing tapping activities. The optimal position of the sensors for the magnetic field emitted and the sensor focal length was investigated using left-hand tapping at 2 Hz. Rest alternating with tapping at 2 Hz was recorded from the scalp surface and repeated in individual tests from 1.5 cm, 3.0 cm, and 4.5 cm away from the scalp surface. The largest voltage response per frequency was from the scalp and 4.5 cm away. This implies that the maximum pole of the magnetic field may be at approximately 4.5 cm yielding the relative focal length of the sensor at the distances tested. The scalp measurement included interference for touching hair and its movement and was not utilized for dedicated testing in future tests. These results are pictured in Figure 4.

The sensors’ degree offset relative to the sagittal, coronal, and axial plane were also evaluated as given in Table 1 above with testing in the sagittal plane which is described later in the manuscript. Due to the above results, it was considered that since the amplitude was greatest at 4.5 cm, the sensor may have maximum theoretical sensitivity at each multiple of 4.5 cm. The test was then repeated at the sensors 4.5 cm from the skin with the angle to the sagittal plane changed 12 degrees. The change in angle increased sensor B319 voltage output by 30-fold and changed the morphology of the waveform slightly which may correspond to a different cell group measurement or altered dipole approximation.

**Figure 5:**
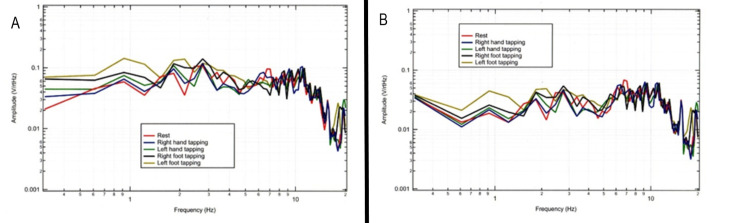
Sensor recordings based on sensor position in relation to the activated motor strip and tapping limb Panel A identifies the measured electromagnetic field by sensors By and B319 when sensing from the posterior position of the helmet directed anteriorly to the motor strip. Panel B demonstrates sensors Bx and Bz identifying signals from the motor strip from the anterior portion of the helmet when directed at the motor strip. Hz: Hertz; V/rtHz: Voltage divided by square route of Hertz

Sensor position was further evaluated in relation to the motor strip as in Figure 5 with a comparison of sensors placed in different locations but oriented towards the motor strip. It was identified that different angles and positioning in relation to the motor strip would yield differing morphologies. It was considered that the sensor appears to measure all frequencies within a cylinder of measurement but identified greater voltages when 4.5 cm away identifying the possible focal point of the EMF. It is assumed that there will be maximum amplitude at multiples of 4.5 cm since the magnetic field expands and contracts along the direction of propagation.

Identification of amplitude changes with tapping

**Figure 6:**
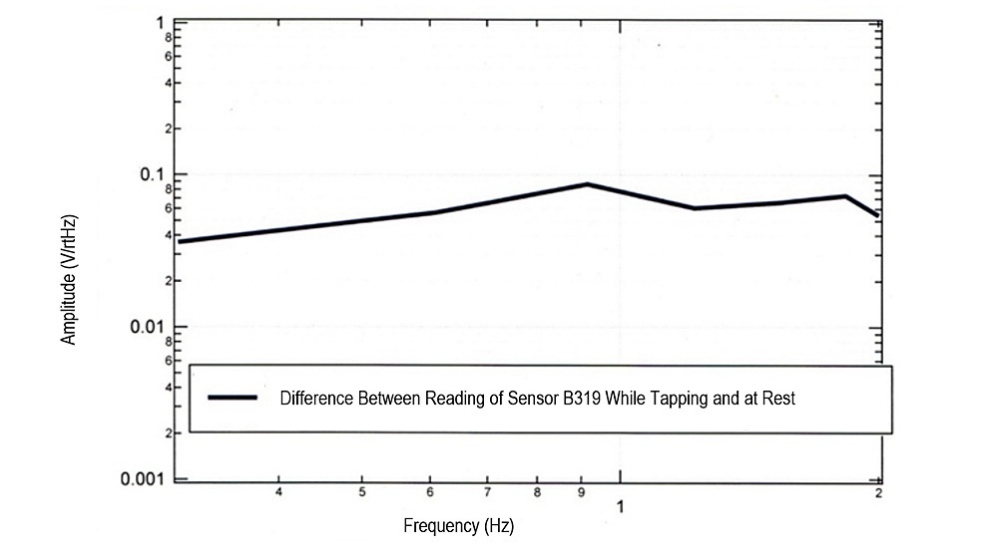
Amplitude changes of Sensor B319 when comparing tapping at rest Summative amplitude changes were noted during tapping exercises with increases in amplitude when performing tapping exercises compared to amplitudes identified when at rest. Hz: Hertz; V/rtHz; Voltage divided by the square root of Hertz

Further analysis was completed to further delineate changes in the measured EMF with tapping activities. EMF was measured during left foot tapping while investigating sensor B319 directed at the right motor strip. The measured EMF was subtracted from the baseline resting EMF generated while foot-tapping was not occurring. The difference was plotted below in Figure 6 which identified a greater than 0 difference with notable peaks consistent with the ability to measure activity secondary to tapping.

**Figure 7:**
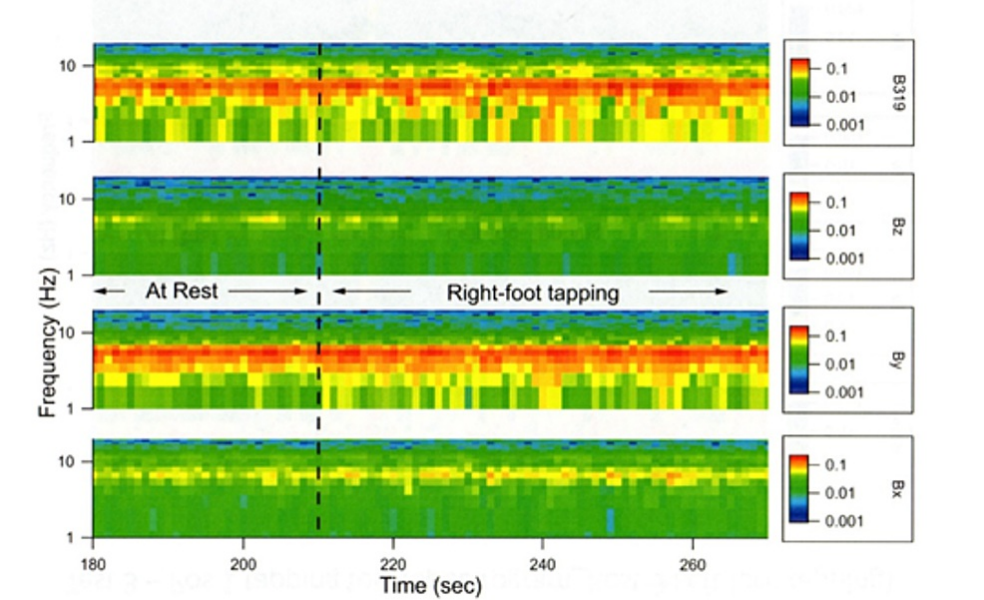
Spectrograph of frequency in real-time The spectrogram shows frequency versus time with sensor frequency response (in Voltage/rtHz) added in color. There is an increase in voltage in B319, By, and Bx at the lower frequencies. sec: Seconds; Hz: Hertz; rtHz: square root of Hertz

Further to evaluate differences between rest and tapping, a spectrogram was constructed utilizing all sensor measurements and the activity of right foot-tapping and rest. An increasing signal was noted as seen by increases in signal intensity especially, in the Bx, B319, and By sensors during tapping compared to rest. This is demonstrated in Figure 7.

Effects of increased cortical activity on tapping through multi-extremity tapping

**Figure 8:**
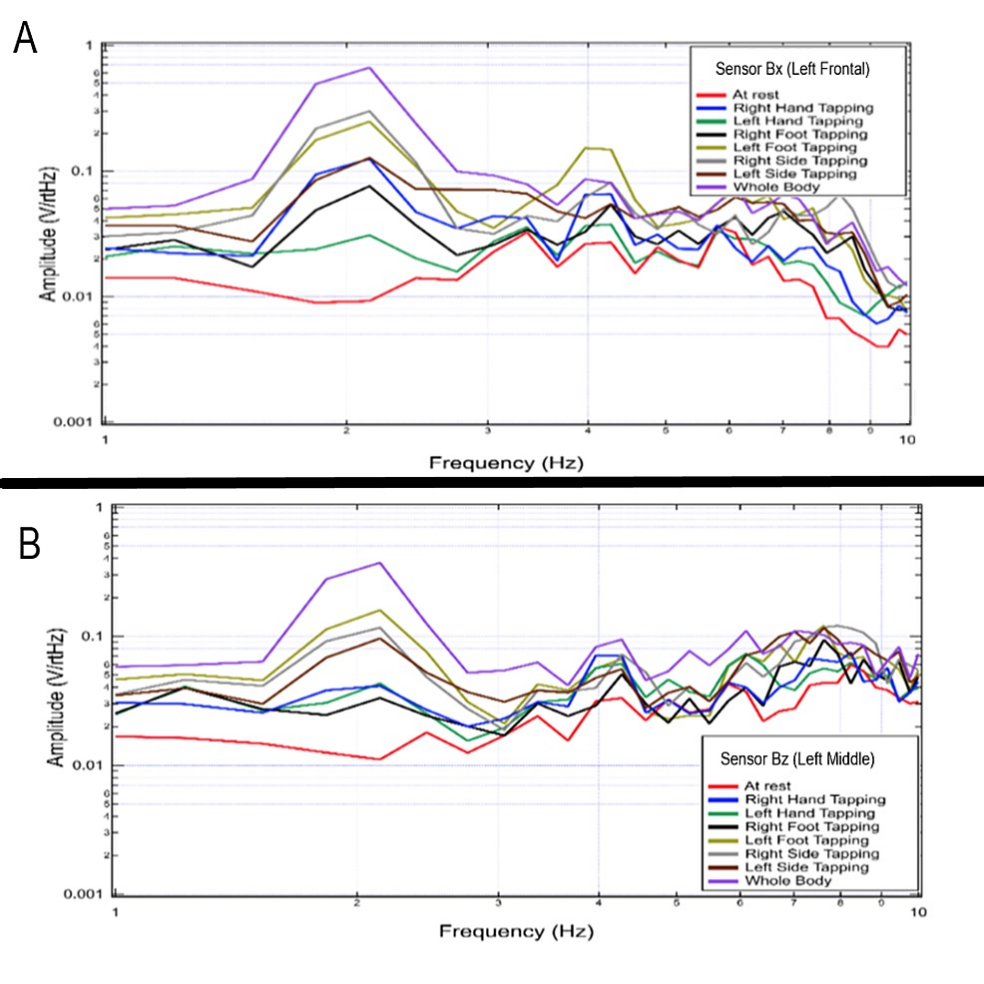
Fourier transformed data at rest and with purposeful movements as recorded by sensors Bx and Bz This figure demonstrates a 20-second interval for Sensor Bx (panel A), corresponding to the sensor of the left frontal region and Sensor Bz (panel B), corresponding left middle region corresponding to the motor strip during right and left-hand tapping, right and left foot-tapping, combined unilateral tapping as well as tapping all extremities. The highest signal change from the baseline resting signal (red) is demonstrated during all four-extremity tapping for both sensors. This maximum change occurs at the 2 Hz frequency corresponding to the tapping frequency. There are also changes from at rest baseline at all other frequencies measured. Hz: Hertz; V/rtHz-Voltage divided by the square root of Hertz

**Figure 9:**
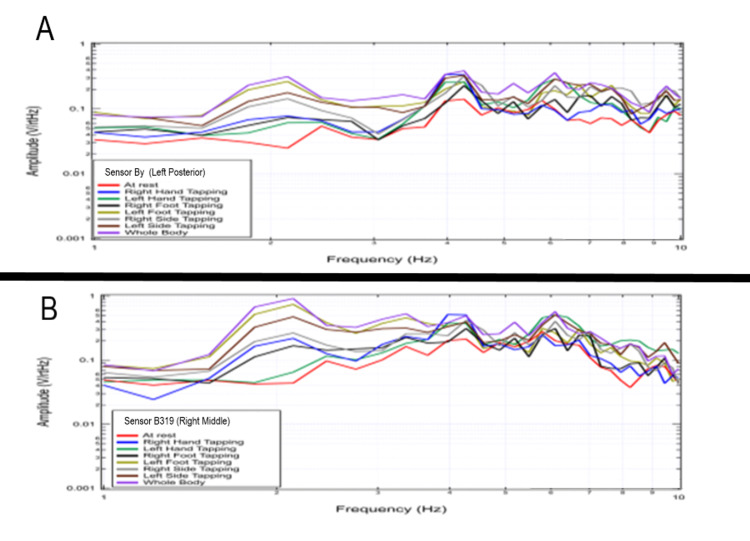
Fourier transformed data at rest and with purposeful movements as recorded by sensors By and B319 This demonstrates a 20-second interval for sensor By (panel A), corresponding to the sensor of the left frontal region and sensor B319 (panel B) corresponding to the sensor located in the right frontal region after performing right-hand tapping, right-foot tapping, left-hand tapping, left-foot tapping, right hand and foot tapping, left hand and foot tapping and tapping of both hands and feet. This again demonstrated a change from baseline rest (red) activity. Hz: Hertz; V/rtHz-Voltage divided by square root of Hertz

During testing of multiple extremities, the shapes of recordings and voltage changes appeared to differ based on sidedness and the location of the sensor. This appeared to correspond with designated activated cortical areas and differed from the baseline brain EMF generated during rest. Changes in voltage appeared greatest at 2 Hz with additional changes in voltage from 4-10 Hz. This was further investigated to evaluate the effects of differing limb activities. Sensors were oriented in standard positioning using the non-shielded helmet and a subject performed repetitive tapping exercises at 2 Hz interspersed with rest using right-hand tapping, left-hand tapping, right-foot tapping, left-foot tapping, right-hand and right-foot tapping, left-hand and left-foot tapping and then tapping with all extremities. The largest changes in amplitude were identified when tapping all extremities but changes were identified from baseline rest activity with all activities. This is represented graphically in Figures 8, 9.

**Figure 10:**
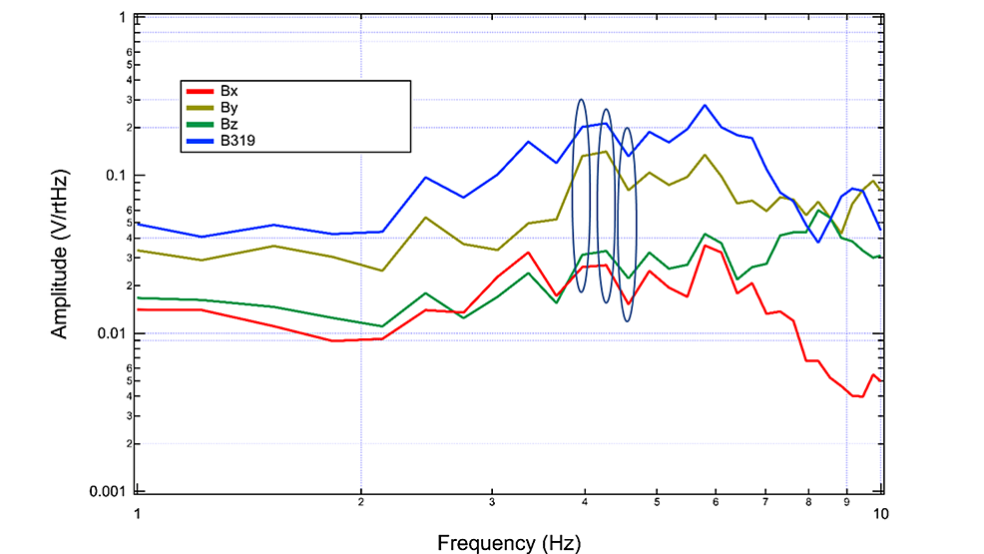
Comparison of sensor readings during rest activity Similarities are identified using ovals on the graph during the rest phase. Sensors are denoted as in the graphical legend with sensor Bx in red, sensor By in yellow, sensor Bz in green, and sensor B319 in blue. Hz: Hertz; V/rtHz: Voltage divided by the square root of Hertz

Sensor reliability was investigated using the rest phase. To do so, sensors were confirmed to be oriented as in Figure 1 above. This was followed by a recording of rest phase with all sensors. The recorded transformed data was plotted in Figure 10. Similar amplitude peaks and valleys were identified during rest by all sensors. Similarities were noted between 4 and 5 Hz as noted by the ovals on the graph.

Effects of shielding on measured rest and activity

**Figure 11:**
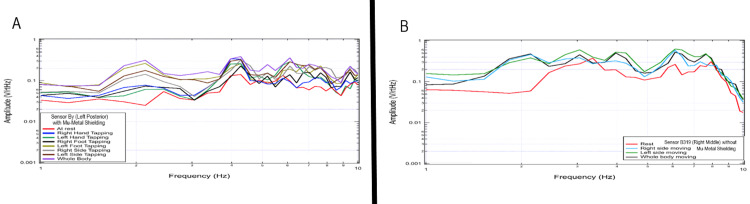
Changes in signal characteristics with mu-metal shielding and without shielding demonstrated by sensors By and B319 Panel A represents results of tapping at 2 Hz with the shielded helmet measured by sensor By in a left posterior location measured a the motor strip and Panel B represents tapping at 2 Hz using sensor B319 without the shielded while located in the right middle (motor strip) location. Hz: Hertz; V/rtHz: Voltage divided by the square root of Hertz

The shielded helmet was then investigated to evaluate the effects of shielding on measured EMF signals during rest activity and tapping exercises. Figure 11 identifies a selected 20-second interval for sensor By (corresponding to the left frontal region) in the top panel with shielding and sensor B319 (corresponding to the left temporal region) seen in the bottom panel without mu-metal shielding during right hand, left hand, foot tapping, as well as whole body tapping. The highest signal was demonstrated during whole body movement for sensor By with shielding, and for left sided movement in sensor B319 (corresponding to the right middle region pointed toward the right motor strip). Changes in amplitude are identified at 2 Hz and 3-4.5 Hz. When evaluating responses without mu-metal shielding there is a noted large amplitude peak from 3-4.5 Hz at rest and during all activities which may correspond to body or room signal which is not seen with the shielding. Shielding using a helmet appeared to improve the quality of EMF signal measured.

Effects of sagittal sensor orientation on measured electromagnetic field

**Figure 12:**
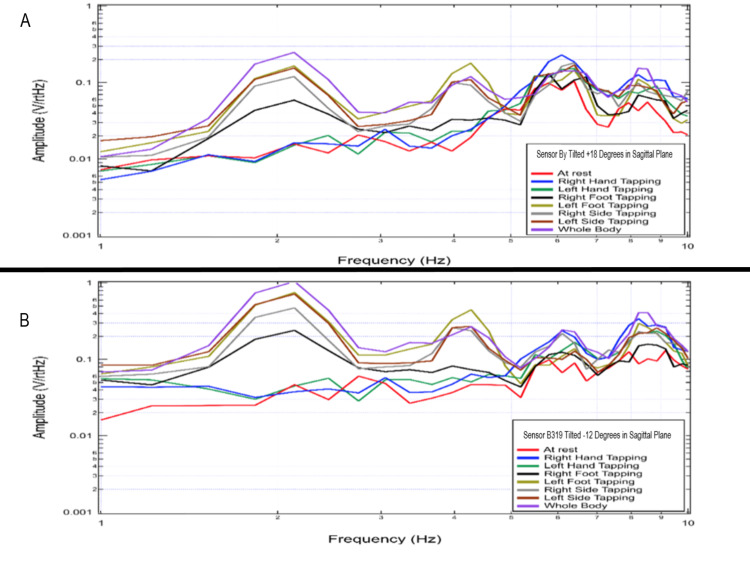
Effects of sensor orientation in the sagittal plane on measured electromagnetic field Panel A represents the transformed data measured by sensor By and panel B represents transformed data measured by sensor B319 after placement as in Figure 3. Sensor By was placed at + 18 degrees in the sagittal plane and sensor B319 was placed at -12 degrees within this sagittal plane. Hz: Hertz; V/rtHz: Voltage divided by the square root of Hertz

The sensors were repositioned into the left middle position in the non-shielded helmet as shown in Figure 3 above. Each sensor was placed at differing angles within the sagittal plane to evaluate the effects of sensor orientation on the ability to sense the measured EMF. As demonstrated in Figure 12, with sensor B319 at -12 degrees and sensor By at +18 degrees, there was a noted amplitude increase in sensor B319 compared to sensor By. It appeared the overall shape of the recording did not differ. This likely represents a more appropriate orientation of sensor B319 in a more perpendicular orientation to the generated magnetic field.

**Figure 13:**
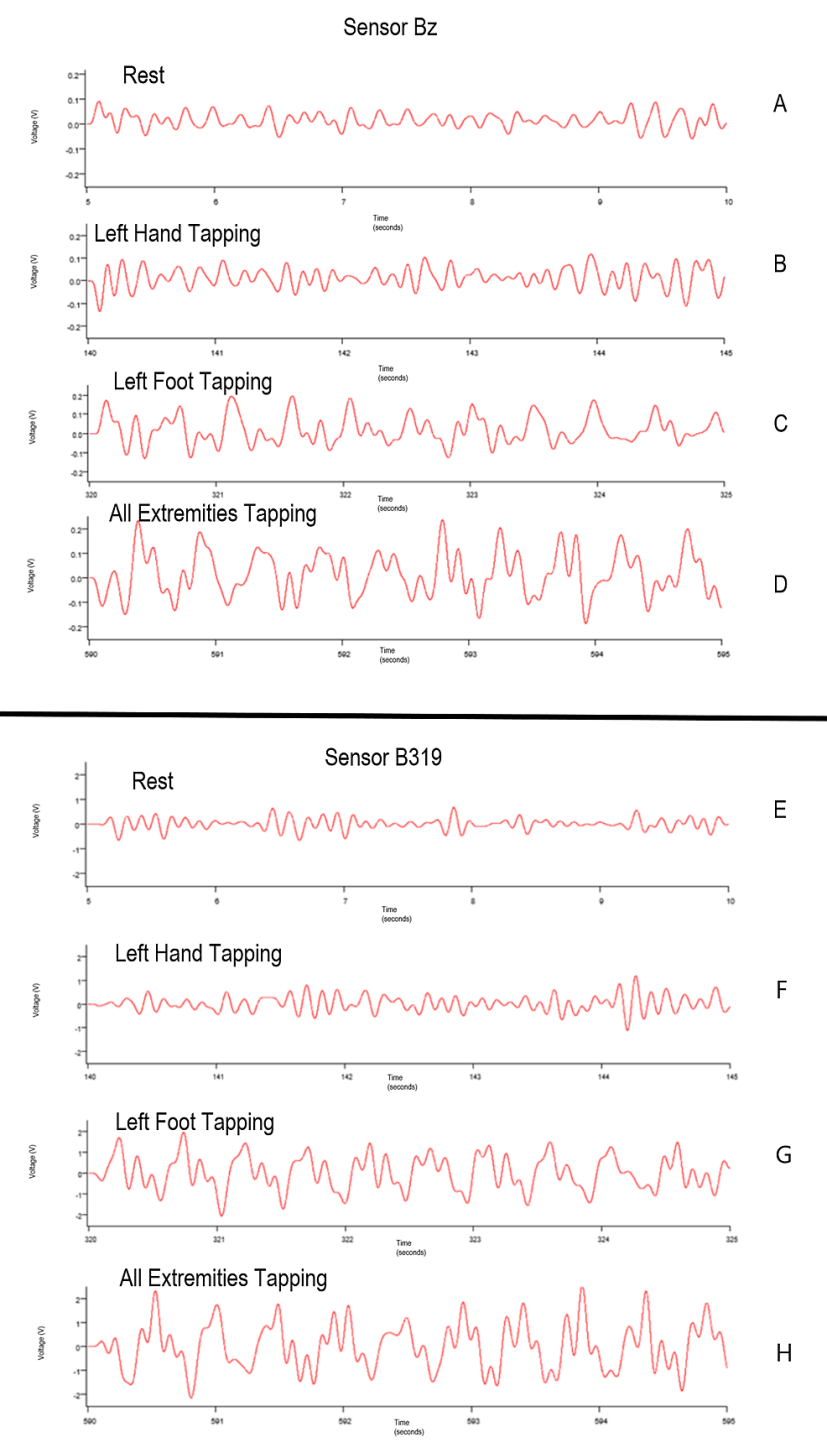
Time-domain plots of voltage measured by sensors Bz and B319 in real time during multiple tapping exercises The overall time-domain plots of the voltage generated through sensors Bz (panels A-D) and B319 (panels E-H) were plotted during rest, left-hand tapping, left-foot tapping; and all extremities tapping time is plotted on the x-axis and voltage on the y-axis.

Using the repositioned sensors within the left middle position over the left motor strip, sensor Bz was placed 12 degrees positive in the sagittal plane from a perpendicular position to the head and sensor B319 was 12 degrees negative to the perpendicular position. Overall voltage sensed by each sensor was evaluated in real time as seen below in Figure 13. It was identified that the Bz graph plotted voltage from -0.2 to +0.2 V and the B319 graph plotted voltage from -2 to +2 V. As more voltage was measured based on the orientation of the B319 sensor, the orientation of sensor B319 may yield a more appropriate position for recording the generated EMF. Though overall waves demonstrate uniformity within timing and location of peaks that identity increases in signal during activity, subtle morphology differences were noted within the different panels identifying that position may be critical for identifying full activity and for future attempts at localization. 

Evaluation of micromovement with a gyroscope to investigate effects on measured electromagnetic activity

**Figure 14:**
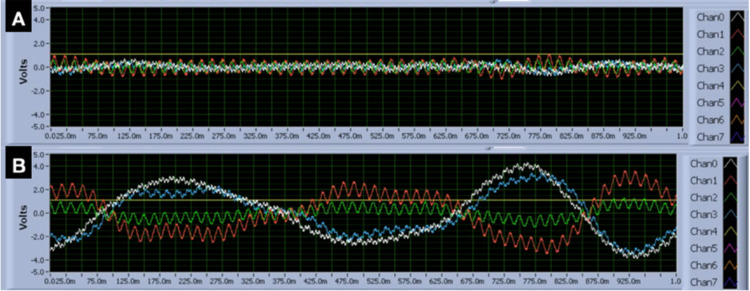
Real-time voltage for the gyroscope and each sensor during inactivity and head-bobbing while tapping at 2 Hz The time-domain during inactivity (panel A) versus an intentional 2 Hz head bobbing phase (panel B) during a tapping experiment. Intentional head bobbing preserved wave shape but dramatically altered voltage changes both positive and negative on the real-time voltage over time recording. In (panel A), there is no significant oscillation of waveforms or position while head-bobbing altered activity. Channels 0-3 demonstrate output from the sensors while channel 4 represents the output of the gyroscope. The green line is a constant 1 V output set as a baseline. Chan: Channel; Volts: Voltage; m: Milliseconds; Hz: Hertz

Identification of whether artifact from kinetic energy from movement played a role in regards to measured signal was critical as the sensors are extremely sensitive devices. The 2 Hz movement demonstrated a specific sinusoidal wave as seen in Figure 14. Further, the data in Figure 14, demonstrates that intentional head movement (Figure 14B) results in significant voltage change, wave propagation, and distortion. This same voltage change was not seen in the stimulus recordings with finger tapping. Therefore, finger or foot 2 Hz tapping does not likely generate enough head movement to affect the recording. Further, an attached gyroscope did not identify significant movement at 2 Hz during pre-described tapping activities. Thus small micromotions did not appear to affect measurements but macromovements of the head may induce interference.

Reproducibility of the sensed electromagnetic field during tapping exercises

**Figure 15:**
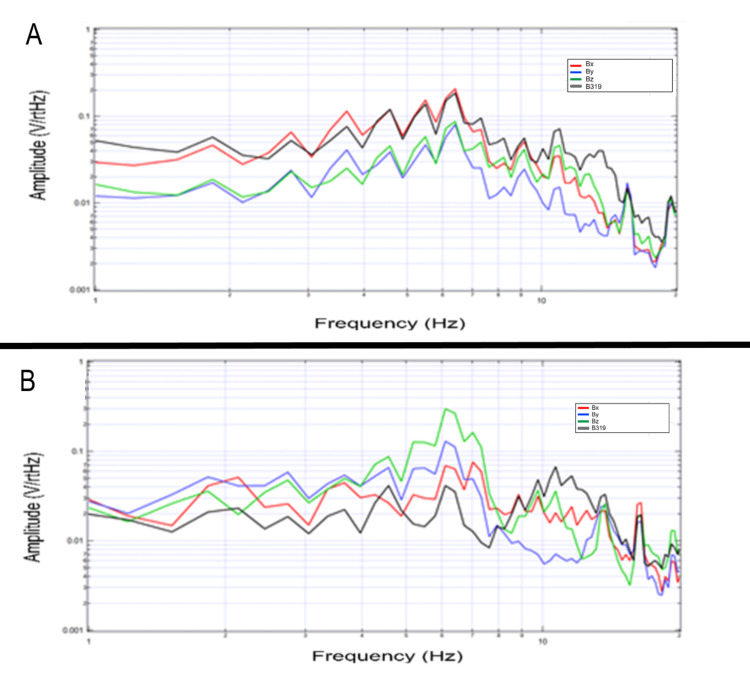
Reproduced electromagnetic field generated by a single subject months apart Panel A identifies the initial measured electromagnetic field signal after tapping at 2 Hz by a single subject with a non-shielded helmet. Several months later the same subject underwent repeated evaluation with the shielded helmet while tapping at 2Hz with results noted in panel B. Sensor Bx is noted in red, By in blue, Bz in green, and B319 in black. Hz: Hertz; V/rtHz-Voltage divided by the square root of Hertz

A volunteer underwent testing several months apart in regards to the standard tapping protocol at 2Hz as seen in Figure 15. Although sensor positioning was measured prior to testing overall positions varied between initial testing and testing with a shielded months later thus there are slight differences in morphology of wave shape but overall there are significant overlaps in peak location during tapping activities. This may correlate to activity from the same subset of cells within the same individual producing the broadcasted EMF and therefore patterns of a specific activity may remain stable with similar amplitude peaks over time within an individual and further identifies reproducibility of measurements.

Tapping at different frequencies

**Figure 16:**
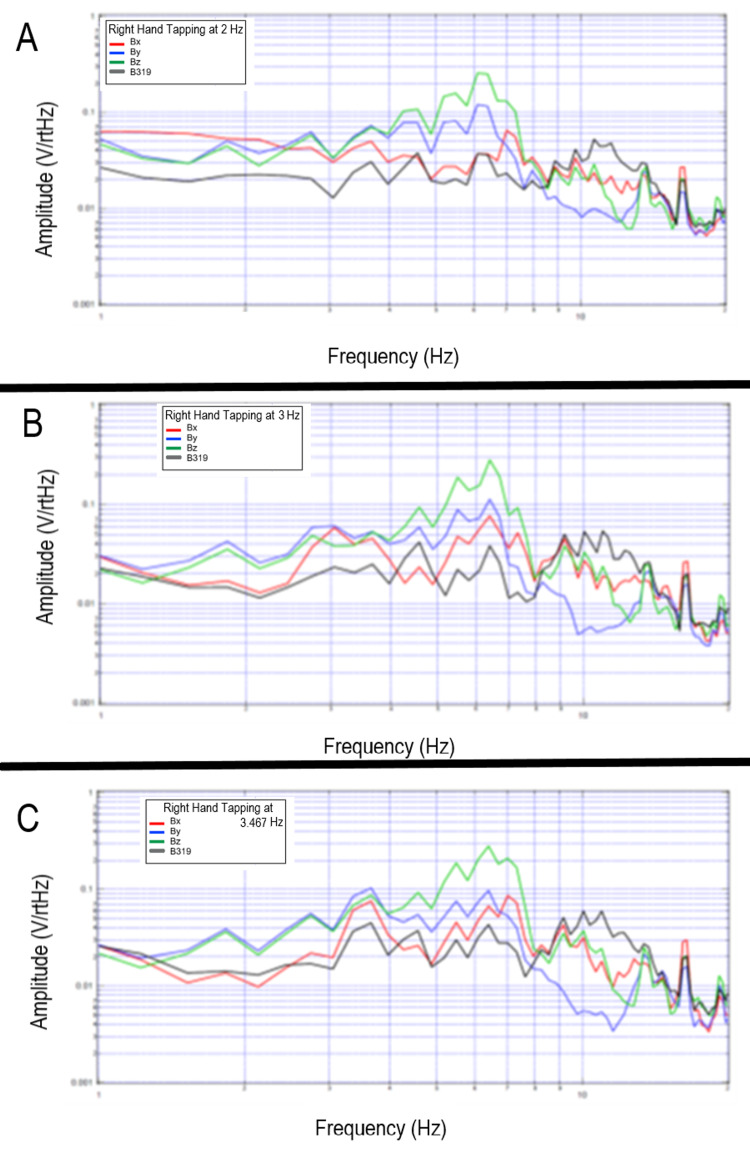
Measured electromagnetic field of a single subject tapping at 2 Hz, 3 Hz and 3.467 Hz Measured electromagnetic field by each sensor was plotted for a single subject tapping at 2 Hz (panel A), 3 Hz (panel B), and 3.467 Hz (panel C). Sensor Bx was denoted in red, By in blue, Bz in green, and B319 in black. Hz-Hertz; V/rtHz; Voltage divided by the square root of Hertz

A subject donned the shielded helmet and was asked to tap at differing frequencies to assess for changes in EMF signaling at different rates and differing cortical activities. It was found that amplitude peaks became sharper in a subject with increasing tapping frequency from 2 Hz to 3Hz to 3.467 Hz. Peaks in frequency locations were overall similar. The changes in peak morphology may be theorized to be due to the coordination of motor activity. The plotted graphs of transformed data are seen in Figure 16. 

**Figure 17:**
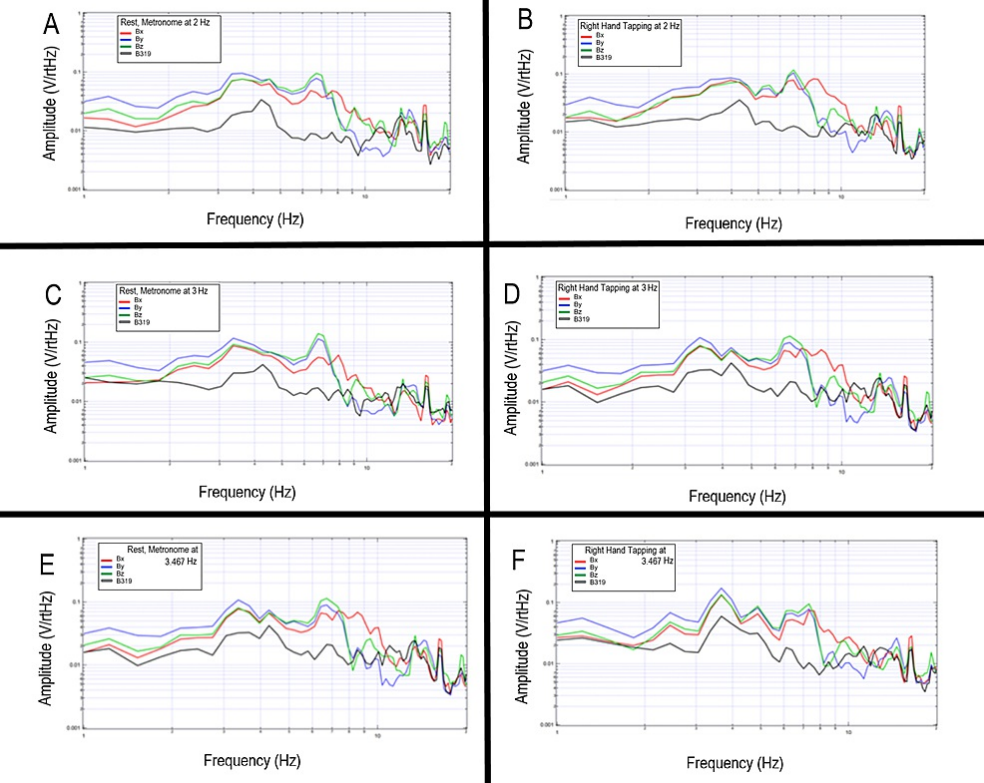
Sensed electromagnetic field of a subject tapping at 2 Hz, 3 Hz, and 3.467 Hz with rest intervals An alternative subject was evaluated completing reproduced tapping activities at 2 Hz, 3 Hz, and 3.467 Hz. Rest intervals were evaluated. Panel A represents tapping at 2 Hz, panel B a rest interval during 2 Hz tapping, panel C tapping at 3 Hz, panel D a rest interval during 3 Hz tapping, panel E tapping at 3.467 Hz, and panel F a rest interval during 3.467 Hz tapping. Sensor Bx is noted in red, By in blue, Bz in green and B319 in black. Hz: Hertz; V/rtHz: Voltage divided by the square root of Hertz

Similar testing with the shielded helmet was completed at rest and with tapping with an additional subject as shown in Figure 17. Differences were noted between rest and tapping at all frequencies with changes within peaks at 2 Hz, 3 Hz, and 3.5 Hz. Morphology was different in this subject than the subject in Figure 16 (above) likely due to slightly differing anatomy and orientation of the magnetic field possibly due to relative differences in skull size and shape as well as subtle differences in relative cortical anatomy. However, the peak locations were similar demonstrating similarities with coordinated complex motor patterns between gross motor activities between individuals.

Multi-subject evaluation of tapping activities

**Figure 18:**
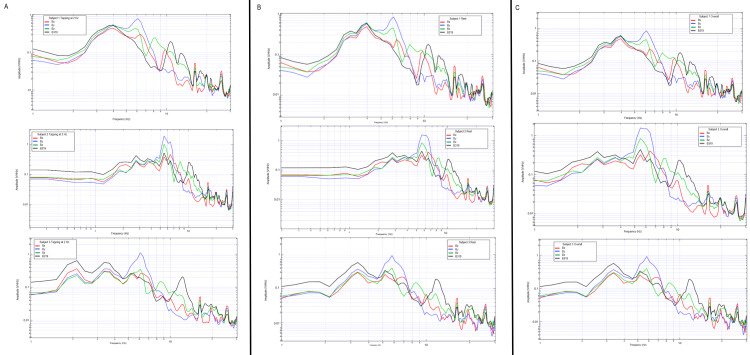
Brain electromagnetic field patterns of three subjects during rest, tapping at 2 Hz, and a plotted combination of rest and tapping Graphical representations of subjects 1-3 are identified in the above figure. Subjects are represented in descending order from subject 1 at the top with subject 3 at the bottom. Tapping activities are seen in the left-most column (column A) for each subject, rest is identified in the middle column (column B) and combined rest and tapping is seen in the right column (column C). Sensors are represented on the graph with red representing sensor Bx, blue representing sensor By, green representing sensor Bz, and black representing sensor B319. Hz: Hertz; V/rtHz: Voltage divided by the square root of Hertz

**Figure 19:**
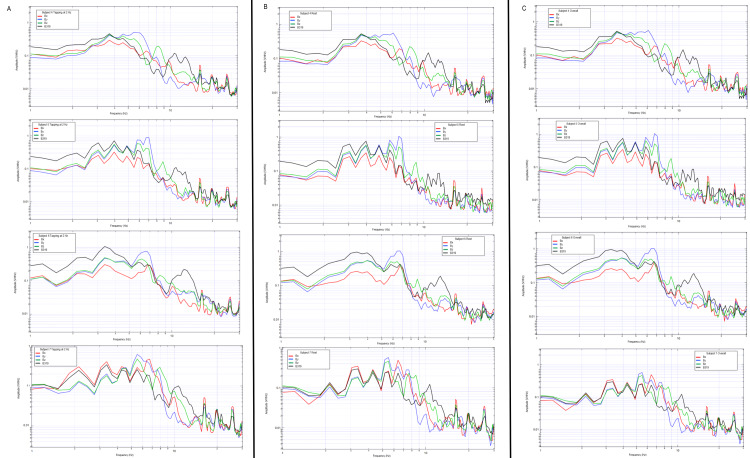
Brain electromagnetic field patterns of four subjects during rest, tapping at 2 Hz, and a plotted combination of rest and tapping Graphical representations of subjects 4-7 are identified in the above figure. Subjects are represented in descending order from subject 4 at the top with subject 7 at the bottom. Tapping activities are seen in the left-most column (column A) for each subject, rest is identified in the middle column (column B) and combined rest and tapping is seen in the right column (column C). Sensors are represented on the graph with red representing sensor Bx, blue representing sensor By, green representing sensor Bz, and black representing sensor B319. Hz: Hertz; V/rtHz: Voltage divided by the square root of Hertz

Additional human subjects were tested with the shielded helmet by completing the repetitive tapping protocol and motor activities as seen in Figures 18, 19. Each individual demonstrated changes from rest to tapping at 2 Hz with unique characteristics but discernable peaks likely representing cortical-based activities for motor activity.

**Figure 20:**
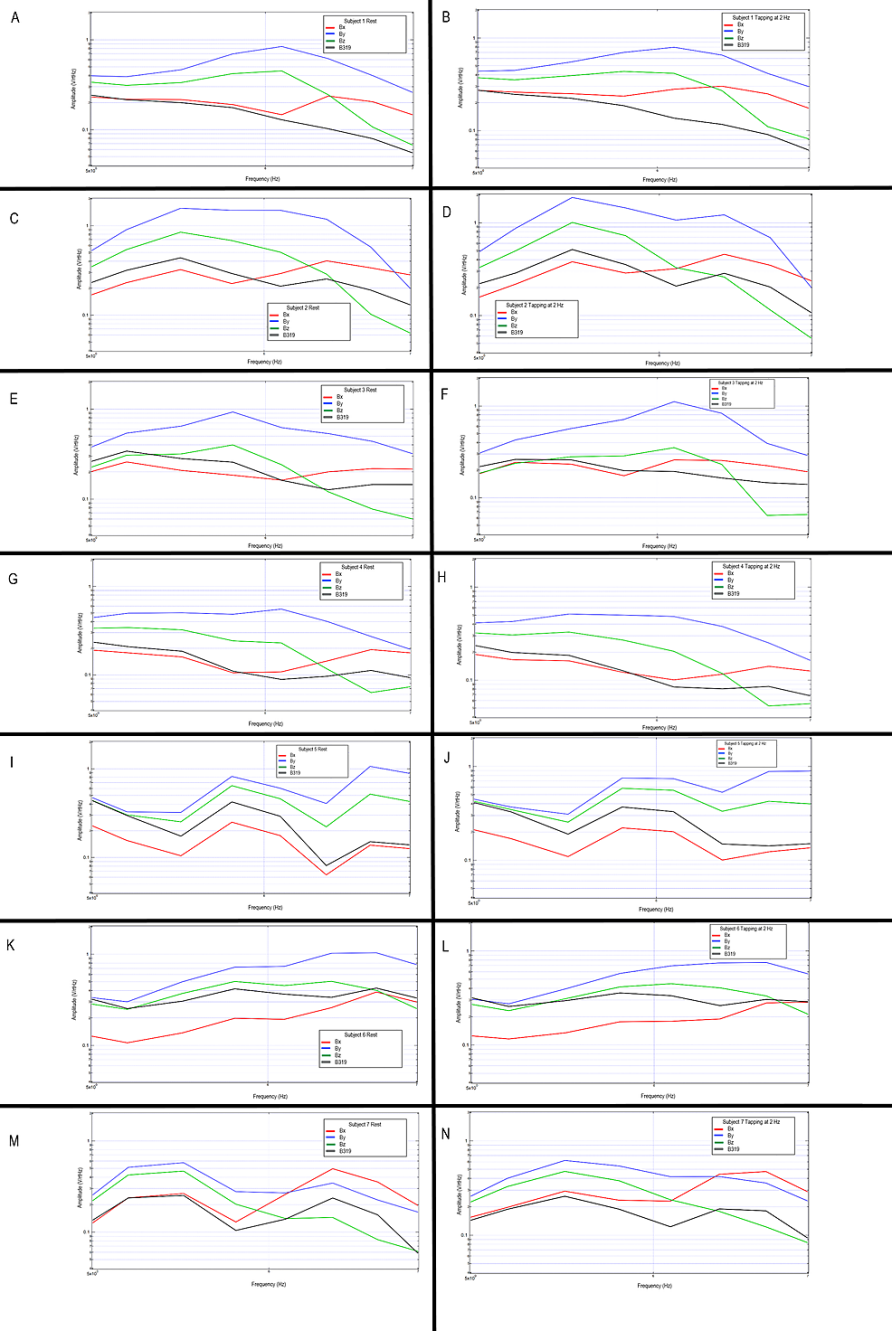
Evaluation of rest versus tapping at 2 Hz amongst seven subjects Graphical representations of subjects 1-7 are identified at rest (left column) and tapping at 2 Hz (right column). Subjects are represented in descending order with subject one at the top and subject 7 at the bottom. Graphically the transformed data is identified with the red line representing sensor Bx, blue as sensor By, green as sensor Bz, and black as sensor B319. Hz: Hertz; V/rtHz; Voltage divided by the square root of Hertz

The largest changes between individuals were seen between 5 and 7 Hz. Graphs were generated identifying changes between rest and tapping activities between the seven subjects at these differing frequencies. Each individual demonstrated differences between rest and tapping as well as differences in peaks and valleys within this subset area. This area may represent the region for further investigation for cortical-based activities within the generated EMF from motor activity. The region between 5 and 7 Hz during rest and tapping for each subject is graphically represented below in Figure 20. 

## Discussion

The ability to sense the generation of EMF signals has been documented by Wiginton et al. [3]. Anatomically, normal brain functions have been well described and localized physiologically such as generating and planning movement, processing auditory stimulus, visual imagining, and emotion [5]. Further, summative analysis using Fourier transformed data has been previously demonstrated with these sensors but has also been utilized in spectral EEG analysis [3,6]. Thus, this methodology of waveform analysis may be effectively utilized to analyze neuronal activity. By utilizing these sensors approximately 4.5 cm away from the cortical surface of the brain, we were able to reliably identify changes in EMF waves obtained by these sensors in multiple volunteers when participating in known stereotyped motor activity. By directing the sensors anatomically toward the motor strip, it appears possible that we are able to appropriately evaluate and record cellular columns in real-time generating these magnetic fields from a distance in a real-time passive manner in the standard non-clinical, continuous, non-invasive, non-contact, using a small, lightweight, inexpensive helmet.

At first, the shielding was utilized in a mu-metal cube within a Faraday cage. The helmet was then modified to include shielding with mu-metal integrated within the helmet to a helmet roughly the size and shape of a bicycle helmet. Overall, the magnetic field generated by brain activity is generated from activity simultaneously from multiple areas of the brain within both hemispheres. Thus, there is some degree of coordination of function across the brain with and without direct connections. The ionic movement and electrical signals generated are a result of that coordination of function.

Trials without any mu-metal shielding resulted in significant interference from ambient electromagnetic fields, even in a magnetically shielded room as seen in Figure 11. The background body activity did not produce a discernable positive signal in the experimental configuration at 2 Hz but does produce a 0.004 voltage change between 3 and 4 Hz. When the reproducible testing protocol of rest, alternating with tapping was recorded from the scalp surface, and repeated in its entirety from 1.5 cm, 3.0 cm, and 4.5 cm away from scalp surface the best signal was identified at 4.5 cm as seen in Figure 1. The recording from the scalp surface was considered to potentially be prone to micro-movement interference from contact from the subject's hair. Despite this concern, evaluation of gyroscopic testing in micromovement during tapping did not affect the generated EMF when at further distances. Further, analysis of results from the recordings most proximal to the scalp did not demonstrate the largest response, possibly due to a grounding effect. Therefore, due to the extreme sensitivity of the sensors to record passively in a non-contact fashion from the body or environment, mu-metal shielding of the head and sensor placement at intervals of approximately 4.5 cm was required.

Recording of activity occurs in real-time. However, these sensors are non-contact fashion and it appears may represent a deep subcortical brain as during testing with the shielded helmet sensors from the right side that were directed at the left motor strip were able to pick up identified EMF traversing through to the contralateral hemisphere. Although the waves demonstrate the 2 Hz nature of the recording for tapping, differences in morphology were identified in a different area of the brain that is activated during the stereotyped activity. The summative measurements of this Fourier transformed motor-based data are represented above. Further, this stereotyped activity differed from the rest as seen by the differing morphologies above. This difference in morphology and voltage may also be a factor of the sensor positioning, sensor angle, distance from the surface, cortical location of the generated activity, and based on the individual as noted in Figures 12, 13.

The graphs demonstrate all changes within the chosen frequency and voltage displayed. Each sensor records the voltage/rtHz dependent upon the sensor’s orientation to the magnetic field and distance away from the source, thus the voltage accumulated and measured in each bin is dependent on a specific sensor orientation. Therefore, only neuronal sources generating a magnetic field can be identified and located by multiple sensors as they are likely to have the greatest summative activity. As sensors traveling from the contralateral side were able to identify summative voltages, these measurements of the magnetic field may be measured from any depth in the human head. As the magnetic field is maximized at its perpendicular, ensuring the sensor is perpendicular to the desired tissue can be utilized to maximize measurement and may be utilized in future experiments for triangulation and localization by identifying the maximal dipole [7]. Correspondingly, the sensor most stimulated depends on which limb is thought to move corresponding to the brain motor homunculus and brain region. Although differences were noted between individuals being tested, a measured EMF from an individual with the same configuration of sensors doing the same stereotyped activity did yield waves in similar morphology and peaks over time delineating an appropriate measure of generated EMF as seen in Figures 18-20.

Distortion of the magnetic field being generated by the brain as it exits the brain traverses through the dura, bone, and skin was also considered when initial results were obtained. However, it has been demonstrated that biological tissue has minimal to no distortional effect on magnetic fields [8].

Using a shielded helmet, tapping and rest-activity were readily identifiable and demonstrated unique characteristics based on the frequency of the motor-based activity as seen in Figure 17. Further with the shielded helmet, the magnetic field recording varied based on the distance from the source, the angle to the magnetic field, and recorded brain activity simultaneously at multiple frequencies in small focal regions as demonstrated by measured changes in recorded EMF with sensor trajectory adjustments. 

These magnetic fields were also identifiable in multiple different subjects participating in stereotyped motor activities with a shielded helmet. Although morphologic characteristics differed between participants, likely secondary to anatomic differences in both connectivity and physical anatomy, it was seen that reproducibly measured signals were obtained through repeated testing of stereotyped motor activity between rest and tapping. Therefore, these motor activities can be reliably measured using this methodology. Future studies may wish to investigate other cortical functions as well as investigate applications of this mapping into clinical practice to better map and define these waves beyond identification in the individual. Additionally, as demonstrated with these subjects, a frequency between 5 and 7 Hertz may be further investigated as a region of interest in motor-based activities due to variations in measured EMF as seen in Figures 18-20. Further, if the validity of these sensors is verified in additional studies and with additional activities it may be possible to use machine learning to develop algorithms to utilize generated EMF for functional mapping. The mapping may be useful to understand anatomic differences between subjects which may be used in surgical planning. Furthermore, it may be considered that it is possible that cortical lesions may disrupt the normal functioning of the cortex and then may alter electrochemical signaling. These alterations may then affect the individual's EMF and thus if normal EMF ranges or values can be established between subjects for normal activities, the application of this helmet and sensor technology may have some diagnostic use for the evaluation of intracranial pathologies.

Limitations

This project was conducted with novel sensors in a non-contact manner and uses novel techniques to measure EMF. Limitations to this project are that the results of this project were not compared to current technologies; however, current technologies of MEG and EEG differ from the proprietary nature of these sensors limiting comparability. Further studies may be utilized to compare functional testing and activities and correlate it with these sensors, however, this may be limited due to the interactivity of ferromagnetic materials providing shielding within this study and established imaging modalities such as functional MRI. Larger sample sizes can be utilized in the future to further assess the reliability and evaluate for patterns and characteristic similarities between subjects in regards to motor activities. 

## Conclusions

Non-contact non-invasive sensors using a shielded helmet may be utilized to reproducibly detect generated motor activities. Micromotion does not appear to affect measurements when sensors are not rested directly on the scalp. Signals are unique to the individual and can be differentiated from baseline non-human activity as well as is identifiably different than activity generated at rest. Further, activities between individuals have underlying commonalities that may be investigated in future studies to correlate the generated EMF with mapping and localization in real-time with non-contact sensors.
